# Increased arterial pressure in mice with overexpression of the ADHD candidate gene calcyon in forebrain

**DOI:** 10.1371/journal.pone.0211903

**Published:** 2019-02-12

**Authors:** Ahmed Elmarakby, Jessica Faulkner, Paramita Pati, R. Dan Rudic, Clare Bergson

**Affiliations:** 1 Department of Oral Biology & Diagnostic Sciences, Augusta University, Augusta, GA, United States of America; 2 Department of Pharmacology & Toxicology, Augusta University, Augusta, GA, United States of America; University of Utah School of Medicine, UNITED STATES

## Abstract

The link between blood pressure (BP) and cerebral function is well established. However, it is not clear whether a common mechanism could underlie the relationship between elevated BP and cognitive deficits. The expression of calcyon, a gene abundant in catecholaminergic and hypothalamic nuclei along with other forebrain regions, is increased in the brain of the spontaneously hypertensive rat (SHR) which is a widely accepted animal model of essential hypertension and attention deficit hyperactivity disorder (ADHD). Previous studies demonstrated that mice with up-regulation of calcyon in forebrain (CalOE) exhibit deficits in working memory. To date, there is no evidence directly connecting calcyon to BP regulation. Here, we investigated whether forebrain up-regulation of calcyon alters BP using radiotelemetry. We found that CalOE mice exhibited higher mean arterial pressure (MAP) compared to tTA controls. Plasma norepinephrine levels were significantly higher in CalOE mice compared to tTA controls. Silencing the transgene with doxycycline normalized BP in CalOE mice, whereas challenging the mice with 4% high salt diet for 12 days exacerbated the MAP differences between CalOE and tTA mice. High salt diet challenge also increased proteinuria and urinary thiobarbituric acid reactive substances (TBARs) in tTA and CalOE; and the increases were more prominent in CalOE mice. Taken together, our data suggest that upregulation of calcyon in forebrain could increase BP via alterations in noradrenergic transmission and increased oxidative stress during high salt challenge. Overall, this study reveals that calcyon could be a novel neural regulator of BP raising the possibility that it could play a role in the development of vascular abnormalities.

## Introduction

Hypertension affects one-third of the United States population and is a leading cause for cardiovascular disease including stroke and cognitive impairment [1]. The correlation between blood pressure (BP) and brain function is well known [2]. For example, recent studies suggest a link between elevated BP and deficits in cognitive and executive functions such as working memory and attention. In addition, several studies highlight a potential relationship between BP and age-related cognitive decline [1, 3–5]. The link between BP and cognition is also apparent in neurodevelopmental behavioral disorders such as attention deficit hyperactivity disorder (ADHD).

Although the chronic use of stimulant medications could contribute to the elevated BP detected in ADHD patients [6], the incidence of hypertension is above average even in ADHD patients who do not regularly take stimulant medication [7]. Furthermore, adults and children with deficits in working memory and attention often develop hypertension [8]. However, it is unclear whether a common mechanism drives cognitive and vascular dysfunction.

The neural component of essential hypertension is frequently assigned to sympathetic 'overdrive' through both peripheral and central neural inputs. A meta-analysis indicates that in about 40% of studies higher BP is associated with elevated circulating plasma levels of norepinephrine [9, 10]. Increased sympathetic nervous activity is a major contributing factor to the pathophysiology of human hypertension and is observed in the spontaneously hypertensive rat (SHR), a commonly used animal model of both essential hypertension and ADHD [11–14]. Drugs that interfere with sympathetic activation remain an important therapeutic strategy for controlling hypertension and its severely debilitating effects on the kidney [15]. Increased sympathetic drive may also increase oxidative stress and inflammation in hypertensive renal disease [15, 16]. For example, salt loading of SHR further aggravates hypertension-related renal injury by increasing oxidative stress and inflammation [17, 18].

While the medulla region of hindbrain is traditionally considered the BP control center in the CNS, recent evidence has implicated forebrain regions that regulate thirst and osmolality in BP control [19]. One gene expressed in forebrain that has not been explored in the context of BP regulation is calcyon. Calcyon, is the mammalian-specific member of a neuron endosome enriched protein (NEEP) gene family [20]. Functional studies indicate that calcyon protein stimulates endocytosis as well as late endosome biogenesis and transport [20–22]. Genetic studies with ADHD subjects and their relatives implicate calcyon in the incidence of ADHD [23]. Calcyon is abundantly expressed in catecholaminergic and hypothalamic nuclei as well as in several forebrain regions including prefrontal cortex (PFC) and hippocampus [21, 24, 25]. The gene is also highly expressed in SHR [26, 27]. Previous studies in CalOE transgenic mice, in which calcyon expression is up-regulated in forebrain, revealed deficits in working memory and inhibitory control behavior [28, 29]. Intriguingly, the deficits could be pre-empted by turning off transgene expression during adolescence which is considered a critical period for development of executive functions [29]. Here, using CalOE mice as a model of ADHD-related deficits, we tested the hypothesis that up-regulation of calcyon influences BP control mechanisms. We found that CalOE mice exhibit increased BP relative to control littermates, and that the BP differences could be exacerbated by a high salt diet challenge. Plasma norepinephrine levels in CalOE mice were also significantly elevated relative to control suggesting that enhanced sympathetic activation could contribute to the increased BP. In summary, our data suggest a novel mechanism by which a gene expressed in forebrain can regulate BP.

## Methods

All procedures with animals were performed in accordance with the Public Health Service Guide for the Care and Use of Laboratory Animals (Department of Health, Education, and Welfare publication, NIH 80–23). All experiments were also approved and monitored by the Augusta University Institutional Animal Care and Use Committee. Twelve to fourteen-week old male CalOE and tTA control mice were used in the current study. CalOE mice are double transgenic carrying 1) a 'responder' transgene consisting of a tetracycline response element (TRE) promoter positioned upstream of an epitope- tagged human calcyon and 2) a 'driver' transgene encoding the tetracycline trans-activator (tTA) protein preceded by CaMKIIa promoter sequence whereas the tTA mice are single transgenic [28, 29]. The CaMKIIa promoter drives transgene expression in hippocampus, amygdala and a number of other forebrain regions including cortex and striatum [30]. Where mentioned, doxycycline (DOX) was included in the drinking water to inhibit the tTA protein.

CalOE and tTA mice were implanted with telemetry transmitters for the continuous monitoring of BP. Briefly, mice were anesthetized via an intraperitoneal injection of ketamine/xylazine before a neckline incision was made for insertion of arterial catheter connected to an implantable transducer (Data Sciences International, St. Paul, MN) to monitor arterial pressure and locomotor activity 24 hours a day by telemetry. The tip of the telemeter catheter was inserted in the aortic arch via the left common carotid artery; the transducer was implanted subcutaneously in the abdomen. The incision was closed with 5–0 nylon sutures. Following surgery, the mice were housed in individual cages with ad-libitum access to food and water. After one-week recovery from surgery, baseline recordings of mean arterial pressure (MAP), systolic blood pressure (SBP), locomotor activity, and heart rate were then taken every 10 minutes continuously with the Data-Quest System (Data Sciences International, St. Paul, MN). Mice were given standard rodent chow (0.3% salt diet) before being switched to a high salt diet (4% NaCl) for up to 12 days.

### Assessment of systolic BP and biochemical parameters

In a separate set of experiments, SBP was measured in the afternoon using the tail cuff pressure method [31]. SBP was measured in both groups of mice before and after turning transgene expression 'off' with DOX treatment (20 mg/ml in drinking water). Mice were placed in metabolic cages for 24-hour urine collection before and after DOX treatment. Protein excretion levels were measured as an early index of renal injury using the Bradford method for protein determination (Bio-Rad, Hercules, CA). Levels of urinary thiobarbituric acid reactive substances (TBARs) (Cayman Chemical, Ann Arbor, MI) were assessed as markers of oxidative stress. Urinary and plasma catecholamine levels were assessed using commercial available 3-CAT ELISA Fast Track kit from Immusmol (Pessac, France).

### Homogenization of renal cortex for protein expression

Kidney cortex was homogenized in ice-cold RIPA homogenization buffer (Sigma-Aldrich, MO) in the presence of protease inhibitors to assess CYP4A, CYP2C44, and soluble epoxide hydrolase (sEH) by western blotting as previously described [32, 33]. The CYP2C44 goat antibody was purchased from Santa Cruz (Santa Cruz, CA); whereas rabbit antibodies for CYP4A and sEH were purchased from ABCAM (Cambridge, MA). The antibodies were detected with a horseradish peroxidase-conjugated secondary antibody and ECL chemiluminescence (Amersham Biosciences, Buckinghamshire, UK). Intensity of immunoreactivity was measured by densitometry, and relative levels assessed after normalizing for protein loading with β-actin.

### Statistical analysis

All data are presented as mean ± SEM and analyzed using two-way ANOVA followed by Tukey's post hoc test for multiple group comparisons or Student’s *t*-test as appropriate. Analyses were performed using Graph Pad Prism Version 4.0 software (Graph Pad Software Inc., La Jolla, CA). For all comparisons, P < 0.05 was considered statistically significant.

## Results

**Fig 1:**
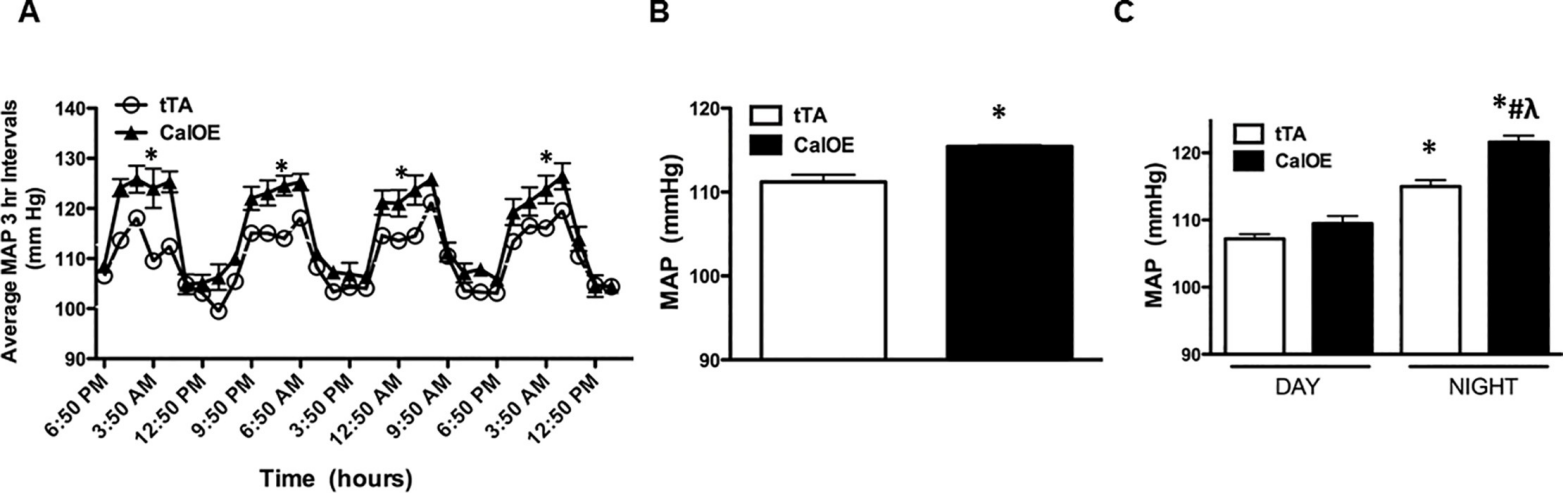
Mean arterial pressure (MAP) of CalOE and tTA control mice fed a standard normal salt diet. MAP of CalOE and tTA mice (A) at 3 hour intervals, (B) during an average 24 hour period, and (C) at night and day across four days (n = 4–5; *, P < 0.05 vs. tTA (daytime); #, P < 0.05 vs. CalOE (daytime); λ, P < 0.05 vs. tTA (nighttime)).

**Fig 2:**
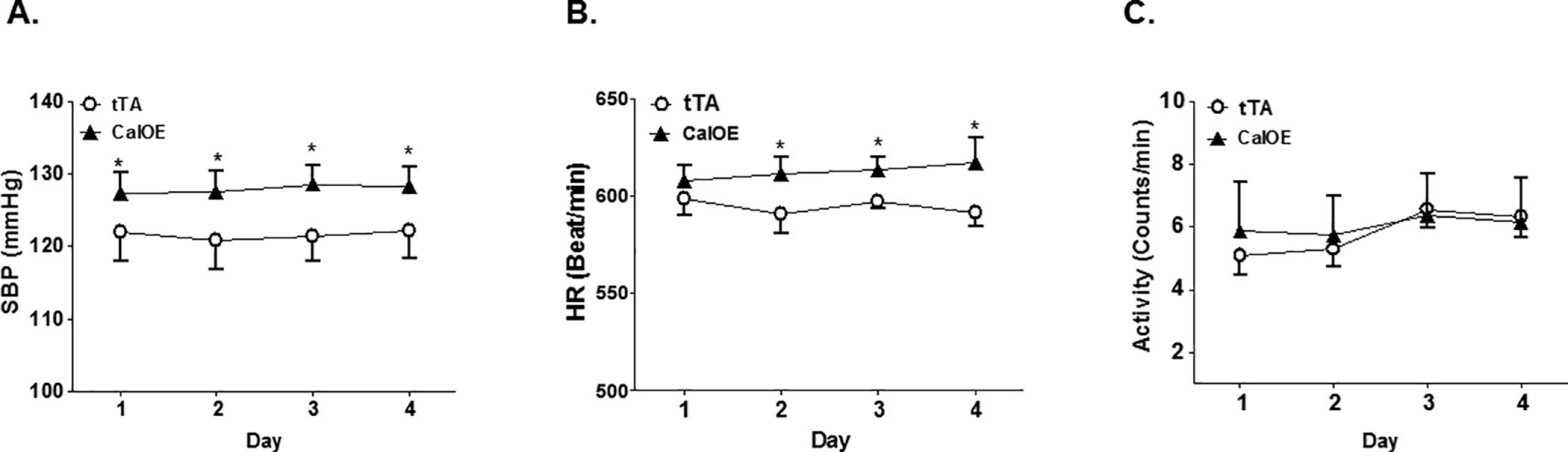
Average 24-hour telemetry data for systolic BP (SBP) (A), heart rate (HR)(B) and activity (C) in CalOE and tTA control mice fed a normal salt diet (n = 4–5; *, P < 0.05 vs. tTA).

**Fig 3:**
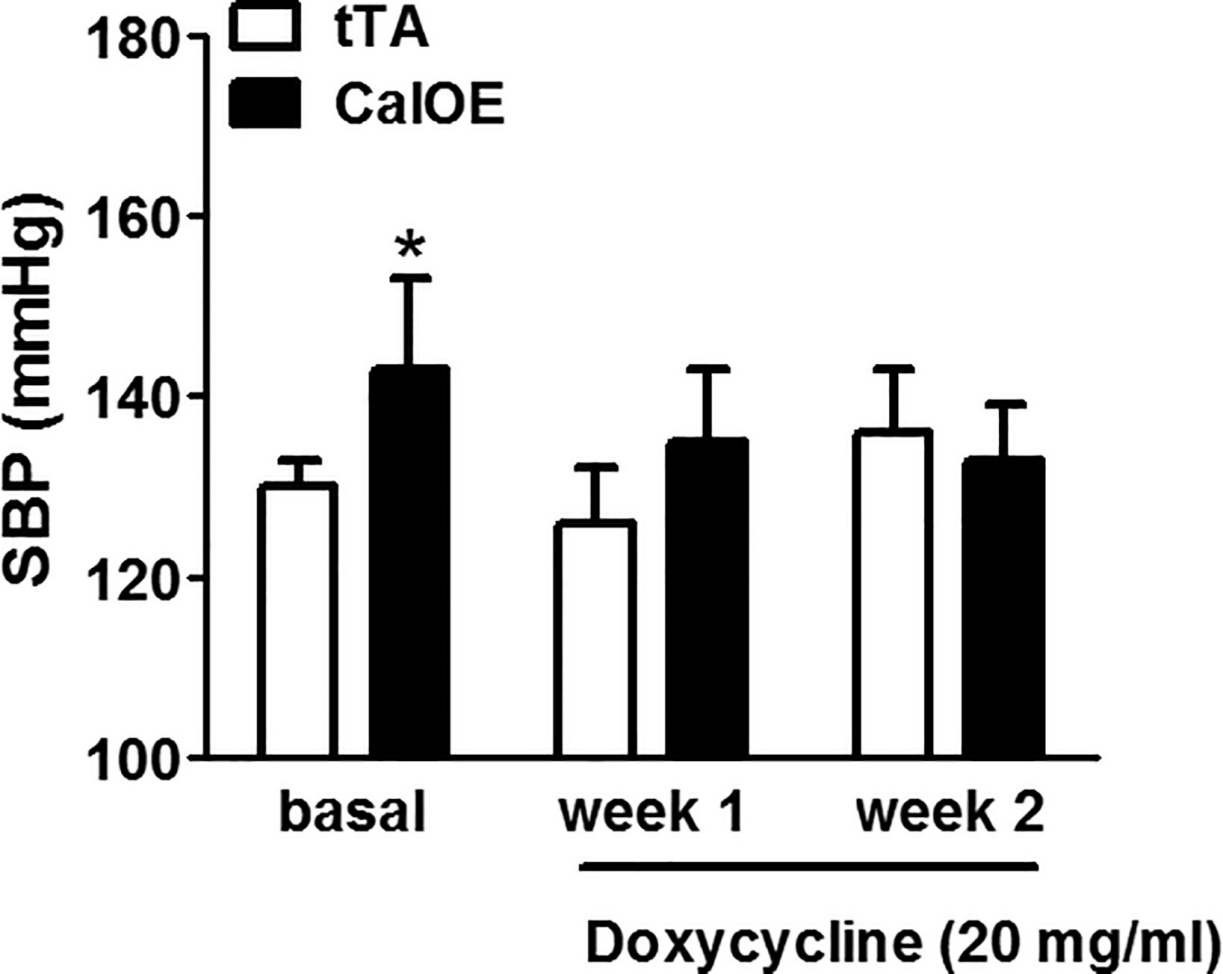
Systolic BP (SBP) measured by tail cuff in CalOE and tTA mice before and after 2-week treatment with DOX. Consistent with telemetry data, SBP was significantly elevated in CalOE relative to controls. However, DOX treatment abolished the difference in SBP between CalOE and tTA mice (n = 5–6; *, P < 0.05).

We used telemetry to assess mean arterial pressure (MAP) in CalOE double transgenic mice using tTA single transgenic mice as controls since initial studies revealed no differences in the systolic blood pressure (SBP) of tTA and TRE-calcyon single transgenic mice. As shown in Fig 1, MAP was significantly higher in CalOE mice relative to the tTA controls (Fig 1A and 1B). The changes in MAP in CalOE were not evident during the day but were prominent at night (Fig 1C). Although CalOE and tTA mice displayed no difference in daily activity, average 24 hour SBP and heart rate (HR) were significantly higher in CalOE mice (Fig 2). A powerful feature of tTA/TRE transgenic systems is that it is possible to turn TRE transgene expression 'off' as well as back 'on' again using DOX to inhibit the tTA protein. Previous findings demonstrated that DOX reversibly silenced calcyon transgene expression in the brain and normalized working memory and fear extinction deficits [29]. Accordingly, SBP was recorded by tail-cuff measurement in CalOE and tTA mice before and after DOX treatment. Consistent with the telemetry data, as shown in Fig 3 basal SBP was higher in CalOE mice relative to controls, and the differences were abolished by DOX treatment.

As DOX normalized SBP in the CalOE mice, we next sought to find out whether DOX might also impact metabolic parameters. While there were no significant differences in body weight or food and water consumption, CalOE mice excreted a lower urine volume relative to tTA controls (0.7 ± 0.18 vs. 1.2 ± 0.07 ml/day, P< 0.05.) As seen with SBP, DOX treatment eliminated the group differences in urine volume (urine volume after one week of DOX treatment was 1.5± 0.30 ml/day in CalOE vs. 1.2± 0.15 ml/day in tTA) (see S1 Table).

**Fig 4:**
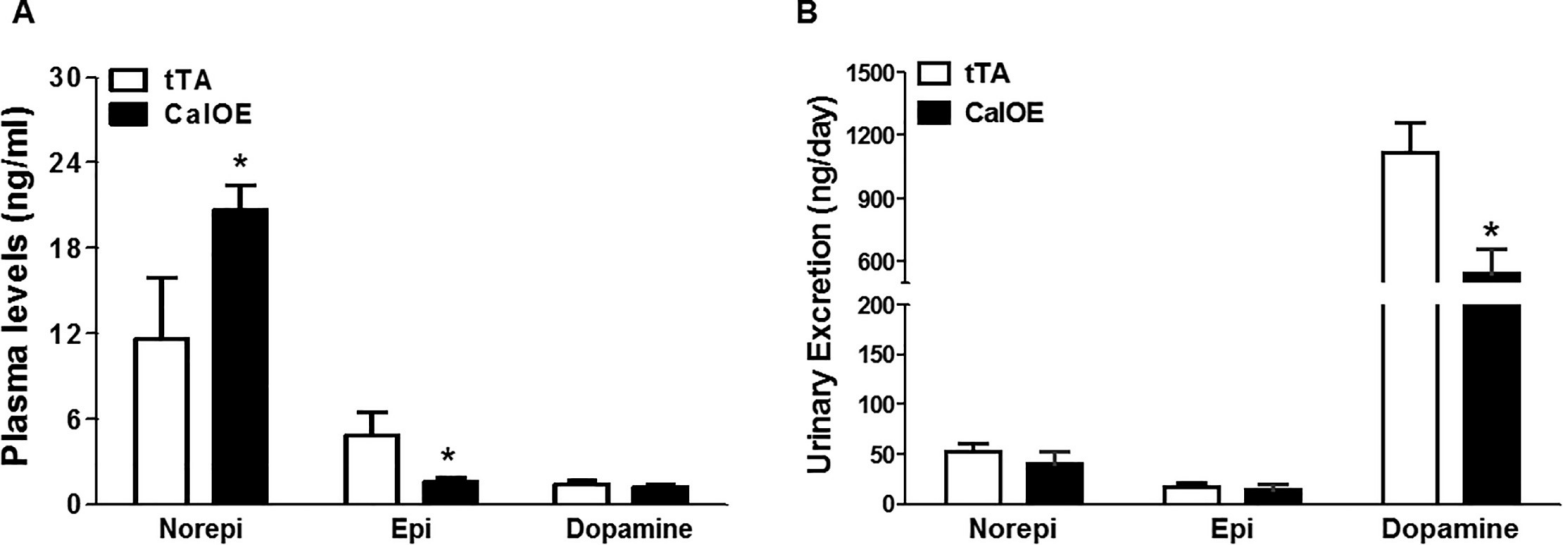
Plasma and excreted catecholamines in urine in CalOE and tTA control mice fed a normal salt diet. (A) Levels of plasma norepinephrine were higher in CalOE mice compared to tTA controls, whereas plasma epinephrine levels were lower. (B) Urinary dopamine excretion levels were lower in CalOE mice compared to controls (n = 5–6; *,P < 0.05).

Catecholamines play a crucial role in regulating BP, especially norepinephrine since it is released form sympathetic nerve endings. We measured norepinephrine, epinephrine and dopamine in plasma as well as urine of CalOE and tTA control mice. While plasma norepinephrine levels were higher, plasma epinephrine levels were lower in CalOE mice compared to controls (Fig 4A). In contrast, levels of dopamine in plasma did not differ between the two groups (Fig 4A). However, urinary excretion of dopamine was lower in CalOE compared to tTA control mice, and no differences in urinary norepinephrine and epinephrine excretion were detected (Fig 4B).

**Fig 5:**
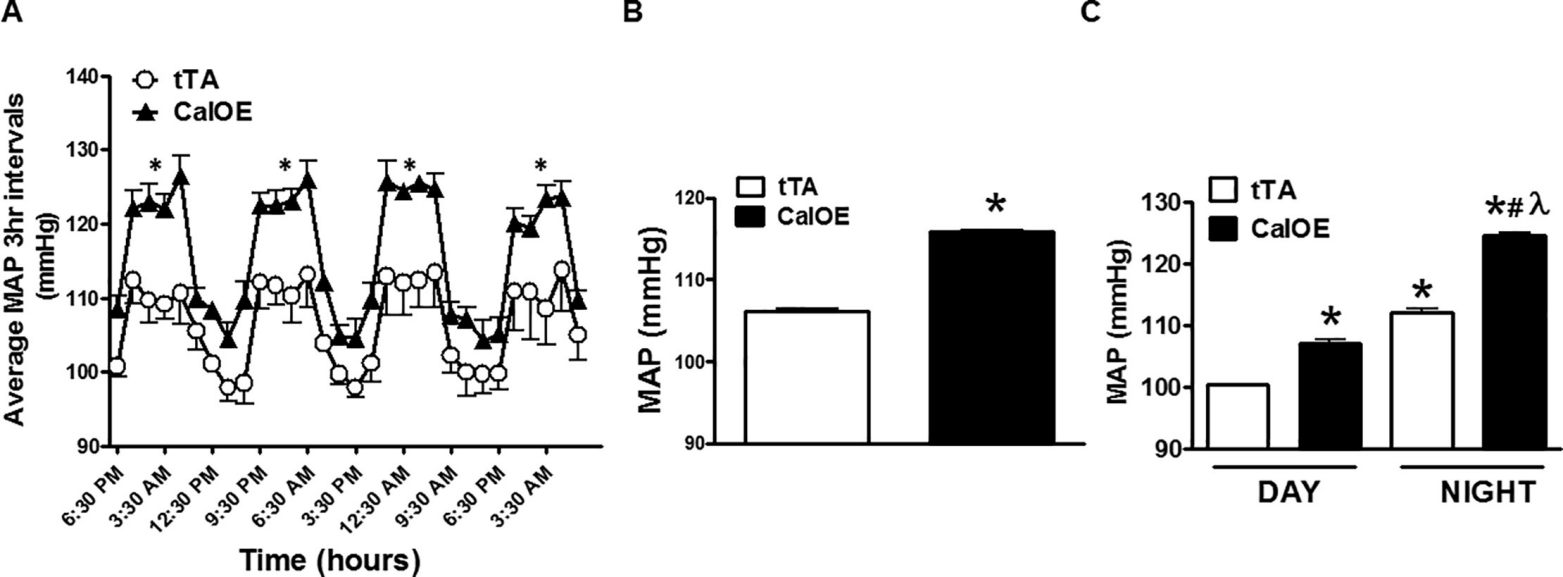
Effect of twelve days of high salt diet on MAP. MAP of CalOE and tTA mice (A) at 3 hour intervals, (B) during an average 24 hour period, and (C) at night and day across four days (n = 4–5; *, P < 0.05 vs. tTA (daytime); #, P < 0.05 vs. CalOE (daytime); λ, P < 0.05 vs. tTA (nighttime)).

We then explored whether increased salt intake might differentially impact BP regulation in CalOE and tTA mice by shifting them from a normal (0.3% NaCl) salt diet to a high (4% NaCl) salt diet for 12 days. As shown in Fig 5A and 5B, high salt exacerbated the differences in BP detected CalOE and tTA mice—the effects of high salt were greater at night than during the day (Fig 5C).

**Fig 6:**
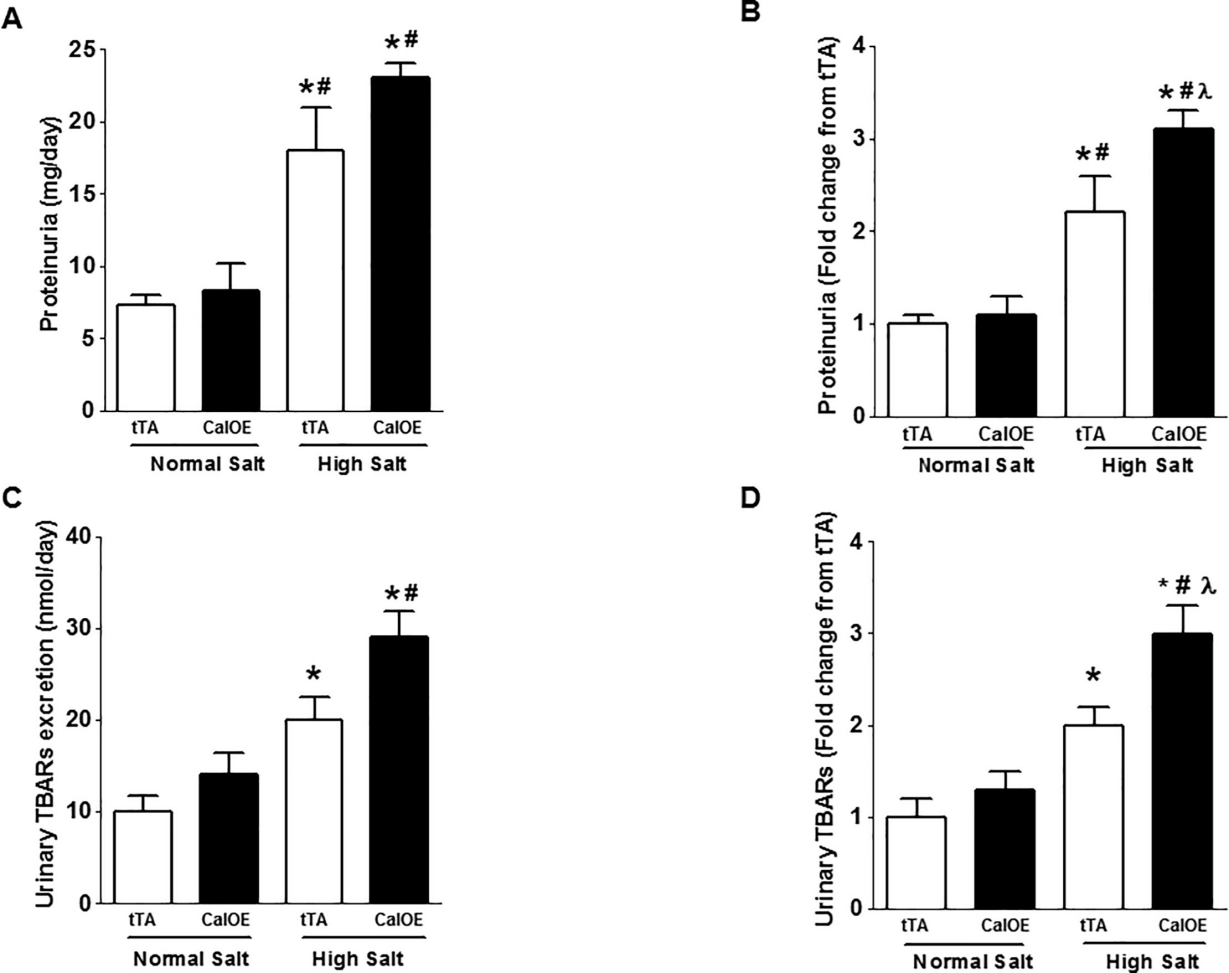
Effect of high salt diet on proteinuria and TBARs excretion. High salt diet treatment significantly increased proteinuria and TBARs in both groups (A, C); the fold change in proteinuria and TBARs excretion was higher in CalOE mice (B,D) (n = 6; *, P < 0.05 vs. tTA (normal salt); #, P < 0.05 vs. CalOE mice (normal salt); λ, P < 0.05 vs. tTA (high salt)).

Besides effects on BP, increased salt intake is known to promote renal injury via increased oxidative stress and inflammation [34]. Therefore, we measured proteinuria and urinary thiobarbituric acid reactive substances (TBARs), both of which are biomarkers for early renal injury and oxidative stress, respectively. No differences in proteinuria were detected in CalOE and tTA control mice on a normal salt diet. While high salt diet challenge increased proteinuria in both groups, the effects of high salt were more striking in the CalOE mice (Fig 6A and 6B). Similarly, there was no group differences in urinary TBARs when on a normal salt diet, whereas urinary TBARs were significantly increased in both groups during high salt intake (Fig 6C). As seen with proteinuria, the fold change in TBARs excretion stimulated by high salt diet challenge was greater in the CalOE group (Fig 6D).

**Fig 7:**
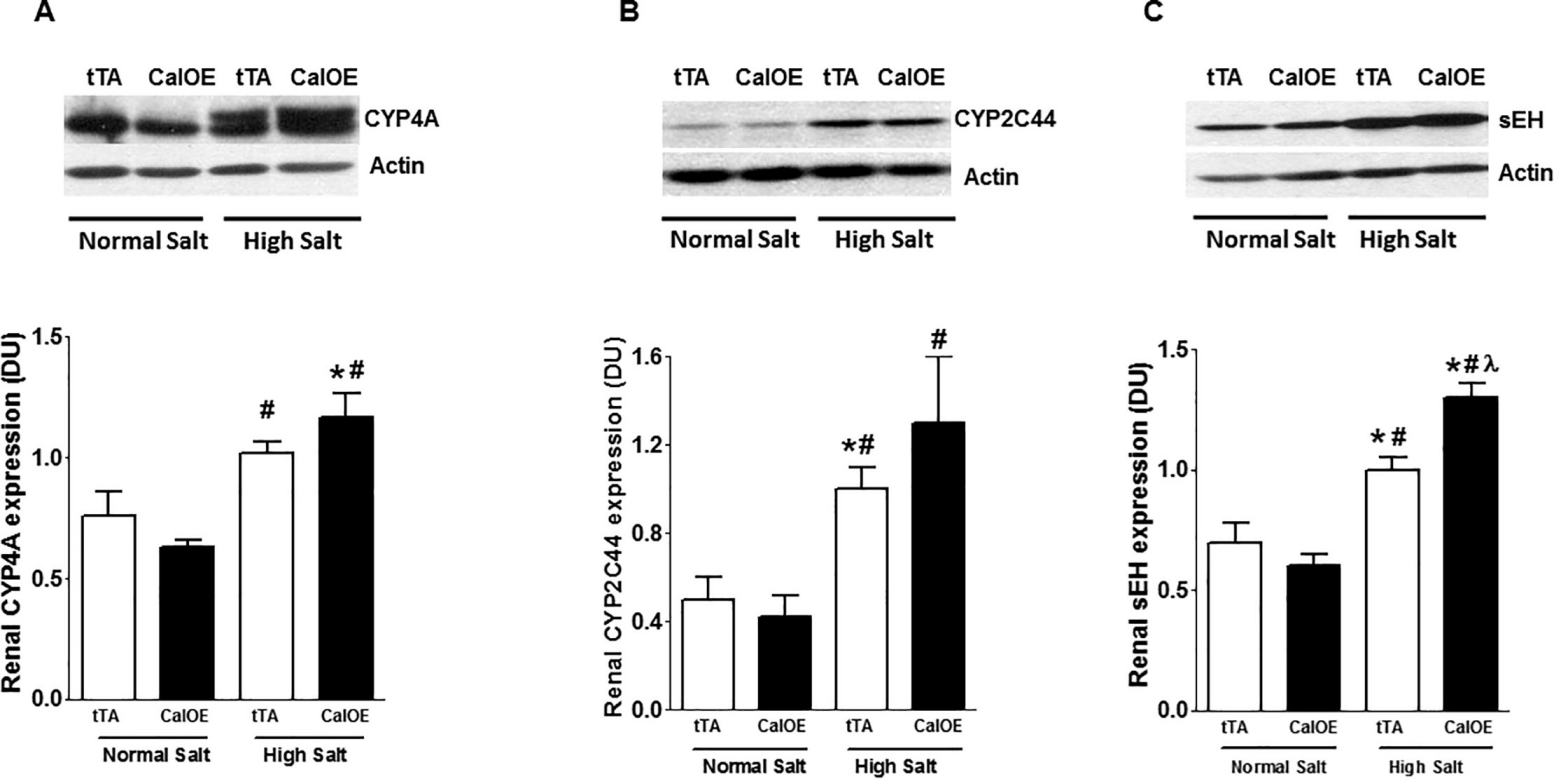
Effect of high salt diet treatment on renal levels of (A) cytochrome P450 hydrolase (CYP4A), (B) cytochrome P450 epoxygenase (CYP2C44) and (C) soluble epoxide hydrolase (sEH). CYP4A is increased in CalOE by high salt diet challenge. CYP2C44 and sEH expression were elevated in both tTA and CalOE mice compared to levels found in mice on normal salt diet. However, only sEH expression was significantly higher in CalOE compared to tTA mice during high salt loading (n = 3; *, P< 0.05 vs. tTA (normal salt); #, P< 0.05 vs. CalOE (normal salt); λ, P < 0.05 vs. tTA (high salt)).

The cytochrome P450 hydroxylase and epoxgenase metabolites, 20-hydroxyeicostetraenoic acid (20-HETE) and epoxyeicosatrienoic acids (EETs), respectively, play an important role in the regulation of BP. 20-HETE and EETs exert vasoconstrictive and vasodilatory effects respectively [35, 36]. However, the vasodilatory effect of EETs is limited by its rapid degradation by soluble epoxide hydrolase (sEH) [36]. Therefore, we measured renal levels of key enzymes involved in EETs and 20-HETE metabolism, CYP2C44, CYP4A and sEH. No group differences in levels of any of these proteins were detected in mice on a normal salt diet. High salt diet significantly upregulated CYP4A in CalOE but not tTA suggesting differential effects on 20-HETE production as a result of calcyon overexpression (Fig 7A). While levels of CYP2C44 and sEH were elevated in both groups on a high salt diet, a larger fold increase in sEH was detected the CalOE group (Fig 7B and 7C). These results suggest that CalOE mice could have lower EETs availability during high salt loading compared to tTA controls.

## Discussion

Mechanisms linking hypertension and cognitive deficits are largely unknown although adults and children with deficits in working memory and attention often develop hypertension [7]. Intriguingly, calcyon is one of many genes with elevated expression levels in SHR which is an experimental model of both ADHD and essential hypertension [26, 27, 37]. Previous studies have demonstrated that up-regulation of calcyon expression in forebrain in the CalOE mice produces deficits in working memory [29]. The current studies indicate that upregulation of calcyon also elevates BP, and that high salt diet exacerbates the BP differences between CalOE and tTA control mice. Importantly, silencing calcyon transgene expression with DOX normalized BP in the CalOE mice implicating calcyon in the central regulation of BP. Our findings revealed that CalOE and tTA control mice differ with respect to a number of key variables including alterations in noradrenergic transmission during normal physiological salt conditions, and oxidative stress during high salt loading—both of which could play a role in the observed differences in BP regulation. In light of our previous work showing working memory and fear extinction deficits in CalOE mice, the findings of the current study raise the possibility that alterations in expression of a single gene could produce both elevated BP and impaired executive functions. More work will be necessary, however, to determine whether the cognitive and vascular abnormalities could derive from a shared mechanism.

Clinical and experimental evidence have demonstrated that noradrenergic transmission plays a major role in the regulation of arterial pressure [15]. Increased sympathetic nervous activity is a major contributing factor to the pathophysiology of human hypertension, and plasma norepinephrine levels are 25–30% greater in patients with essential hypertension compared to age-matched normotensive controls [38]. In the current study, plasma norepinephrine levels were elevated whereas epinephrine levels were lower in CalOE mice compared to controls. Norepinephrine is largely produced by sympathetic postganglionic fibers [39, 40], whereas the adrenal medulla is the main source of epinephrine. Increased norepinephrine in CalOE mice could stem from increased sympathetic activity or from increased synthesis and/or release of norepinephrine. Alternatively, increased norepinephrine in CalOE mice could be due to impaired reuptake by the norepinephrine transporter or degradation by monoamine oxidase (MAO) and catechol-O-methyl transferase (COMT). Along these lines, since sympathetic nerve activity promotes synthesis and release of epinephrine, the observation of lower levels of epinephrine in CalOE mice is somewhat surprising, but could reflect differential regulation of epinephrine reuptake or catabolism, or even alterations in levels of adrenergic receptors or their signaling. Alternatively, other factors including adrenocorticotrophic hormone (ACTH) and cortisol, which regulate epinephrine production and release could also be involved. On the other hand, the elevation and reduction in norepinephrine and epinephrine, respectively, is consistent with the performance of the CalOE mice on a variety of behavioral tasks. For example, while CalOE mice exhibit hyperactivity in a novel environment, they also display anxiolytic behavior by spending more time in areas of testing arenas typically considered more aversive to rodents [28].

We previously reported that CalOE mice display locomotor hyperactivity [28], however, the current telemetry data do not support this conclusion. This discrepancy could be due to the fact that activity in the previous study was measured upon placement of the mice in a novel environment. In contrast, in the current study, activity was measured in the animal’s home cage after more than a week of surgical recovery which presumably allowed time for habituation to the environment. Altogether, our telemetry data suggests that the increased BP and HR observed in CalOE mice could be independent of locomotion.

Our metabolic data revealed that CalOE mice excrete less urine than controls suggesting that upregulation of caclyon might lead to urinary retention. Sympathetic innervation of kidney plays a role in the development of hypertension by enhancing sodium and water retention [41–44]. In humans, renal denervation is a common therapy for treatment-resistant hypertension [45–47]. As CalOE mice exhibit higher BP and plasma norepinephrine levels, calcyon upregulation could stimulate renal sympathetic nerve activation. Interestingly there was no difference in urinary levels of norepinephrine and epinephrine in CalOE relative to control samples, but urinary dopamine excretion levels were reduced in CalOE mice. The discrepancy between plasma and urinary levels of catecholamine could stem from reuptake mechanisms in the kidney, or from their metabolism prior to excretion. Alternatively, there could potentially also be alterations in vasopressin (antidiuretic hormone) in CalOE mice because the CamKII promoter which drives calcyon upregulation is also active in anterior and medial zones of the hypothalamus [48]. Because aquaporin 2 is the primary target for vasopressin regulation of collecting duct water permeability, our future directions will include exploring if vasopressin and/or aquaporin-2 play a role in BP regulation in CalOE mice.

High salt loading stimulates a stressor response that exacerbates the elevation in BP in experimental models of hypertension including SHR [49–51]. Our results showed that high salt diet increased BP differences between CalOE and tTA control mice compared to normal salt diet. The differences were greater at night presumably due to the significant increase in locomotor activity detected in both groups of mice at night. It is worth mentioning that MAP was less in tTA control mice during high salt diet challenge than normal salt diet although these changes with not significant. The drop in BP in tTA and CalOE mice upon salt loading was surprising given previous reports of the effects of a salt challenge in wild type C57BL/6 mice [52]. The regulation of BP is genetically complex, and the role of genes in salt sensitivity is poorly understood [53]. One possible explanation is that the tTA transgene confers some degree of resistance to high salt. Despite this, our telemetry data suggests that high salt diet exacerbates differences in BP between the tTA and CalOE groups, indicating that upregulation of calcyon could confer greater sensitivity to salt.

High salt diet increases proteinuria which could contribute to the progression of renal injury [54]. While there was no difference in proteinuria between CalOE and tTA control mice under normal salt diet, high salt increased proteinuria in the CalOE mice to a greater extent. Elevation in proteinuria and the progression of renal injury in hypertension is strongly associated with increased oxidative stress and inflammation in the kidney. Although the excretion levels of the renal oxidative stress marker TBARs increased in both groups of mice during salt loading, TBARs excretion was elevated to a greater extent in the CalOE cohort. These data highlight the possibility that increased oxidative stress upon salt loading could underpin the elevation in BP and proteinuria detected in CalOE mice.

20-HETE plays dual role BP regulation. In the vasculature, 20-HETE functions as a potent vasoconstrictor to increase BP, whereas in the kidney, it enhances sodium excretion to lower BP [55]. Our data suggests that renal CYP4A expression, the main hydroxylase for 20-HETE production, is increased only in CalOE during salt loading. This result indicates a differential response to salt challenge in CalOE mice at the level of 20-HETE production. On the other hand, we and others previously showed that decreased epoxygenase-mediated EETs production and/or increased sEH mediated EETs degradation plays a role in renal injury in salt-sensitive hypertension [32, 33, 56–58]. In general, increased EETs production during salt-loading exerts a vasodilatory anti-naturetic compensatory response [56, 58], CYP2C44, the main epoxygenase for EETs production in mice, and sEH, an EETs metabolizing enzyme, were up-regulated in kidney in response to high salt loading in both tTA control and CalOE mice. While no group differences in CYP2C44 levels were detected, sEH expression was significantly elevated in CalOE relative to tTA during salt loading. These data suggest that EETs levels could be lower in CalOE mice fed a high salt diet. Lower EETs availability could play a role in the elevated BP differences between CalOE and controls with increased salt intake. EETs are also known to lower oxidative stress [59]; hence, decreased EETs availability could also drive the elevation in TBARs excretion in CalOE mice during salt loading. Whether an imbalance in 20-HETE and EETs levels exists in CalOE mice on high salt diet and if so, whether the imbalance contributes to the elevation in BP in CalOE are questions that remain to be answered.

In summary, our findings provide evidence for the role of a predominantly CNS gene in BP regulation. While the exact mechanism involved remains to be worked out, the present findings suggest that upregulation of calcyon in forebrain is sufficient to elevate BP systemically. Our data suggest that altered adrenergic transmission might drive the elevation in BP; they also suggest that decreased renal EETs availability and/or increased 20-HETE or renal oxidative stress could contribute to the increased difference in BP compared to controls detected in CalOE mice during salt loading. Expression of calcyon is increased about two-fold in brain of SHR animals (5, 16). Since levels of many genes are altered in SHR brain, it seems quite remarkable that elevated BP and executive function deficits are found in mice with upregulation of only a single gene [28, 29]. Taken together, these data highlight the potential use of the CalOE mice in further studies on the central regulation of BP. The CalOE mice could also be a useful model system for exploring mechanisms linking cardiovascular and cognitive functions.

## Supporting information

S1 Table Metabolic cage data from CalOE and tTA control mice fed a normal salt diet before and after DOX treatment (20 mg/ml in drinking water) for one and two weeks (n = 6–8, * indicates P< 0.05 vs. tTA control mice). (TIF)

S1 Fig Representative images for immunohistochemical staining of renal tubular aquaporin-4 in tTA control mice (panel A, brown staining) and CalOE mice (panel B, brown staining) fed normal salt diet at 100X magnification power (n = 3). Panel (C) shows a quantitative representation of aquaporin-4 staining intensity in tTA control and CalOE mice fed normal salt diet (n = 3) using Image J software.
(TIF)

S2 Fig Renal cortical angiotensin II AT-1 receptor expression levels relative to β-actin in CalOE and tTA control fed normal salt diet (n = 4). (TIF)

S3 Fig Raw data for the renal cytochrome P450 epoxygenase (CYP2C44), hydroxylase (CYP4A) and soluble epoxide hydrolase (sEH) expression and their corresponding β-actin in CalOE and tTA control after 12 days of normal and high salt diet treatment. (TIF)
