# Supplementary figures and images for: Increased arterial pressure in mice with overexpression of the ADHD candidate gene calcyon in forebrain
Source: PLoS One. 2019 Feb 12;14(2):e0211903. doi: 10.1371/journal.pone.0211903 (PMC6372185; doi:10.1371/journal.pone.0211903)

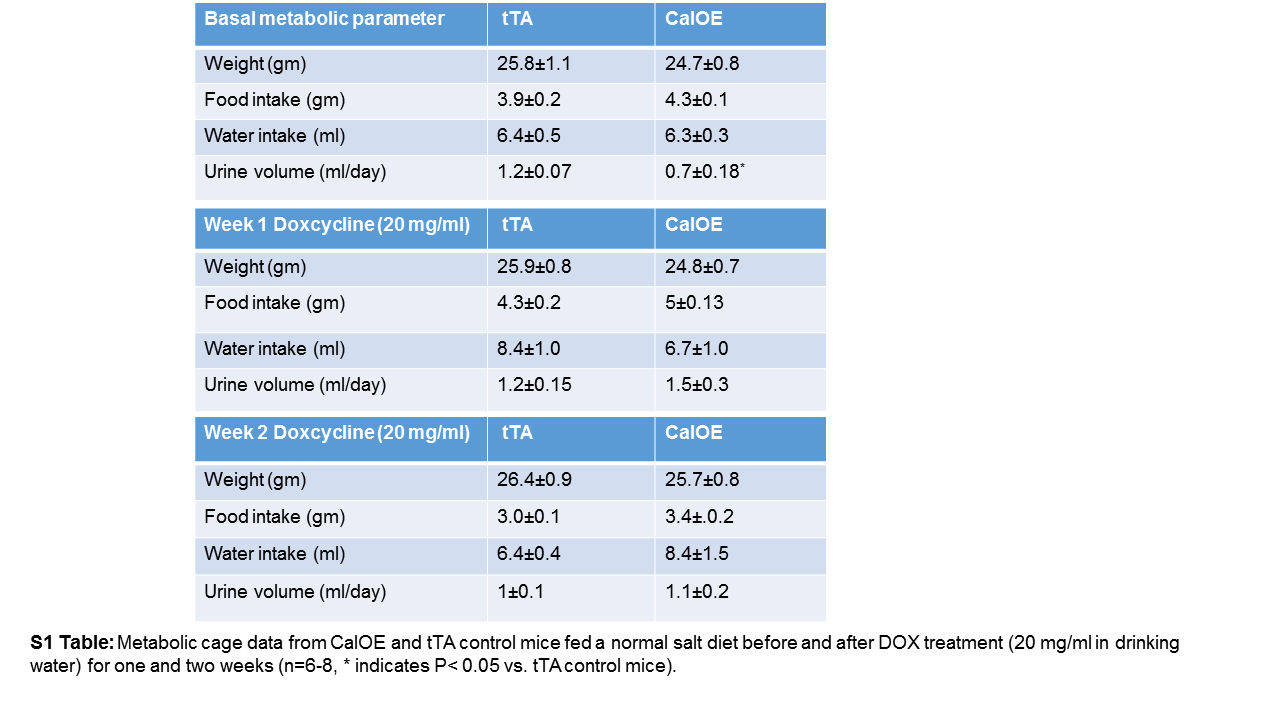

Supplement: S1 Table — (TIF) [file pone.0211903.s001.TIF]

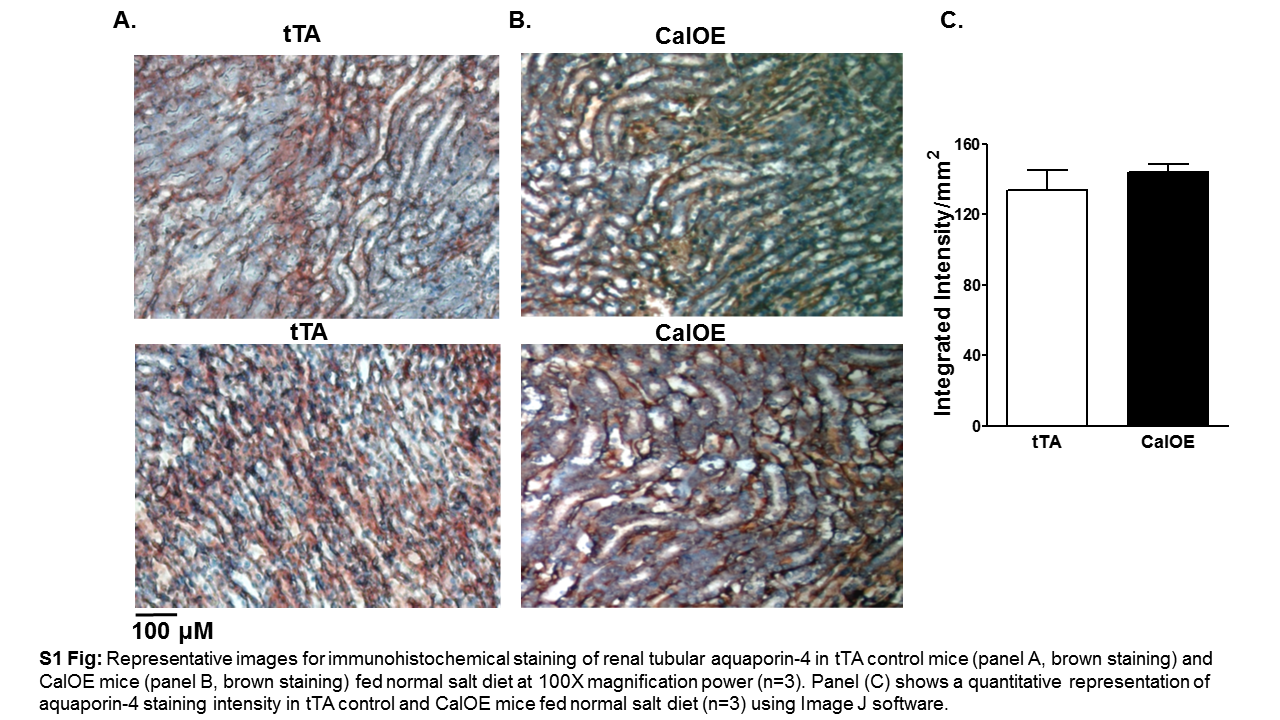

Supplement: S1 Fig — Representative images for immunohistochemical staining of renal tubular aquaporin-4 in tTA control mice (panel A, brown staining) and CalOE mice (panel B, brown staining) fed normal salt diet at 100X magnification power (n = 3). Panel (C) shows a quantitative representation of aquaporin-4 staining intensity in tTA control and CalOE mice fed normal salt diet (n = 3) using Image J software. (TIF) [file pone.0211903.s002.TIF]

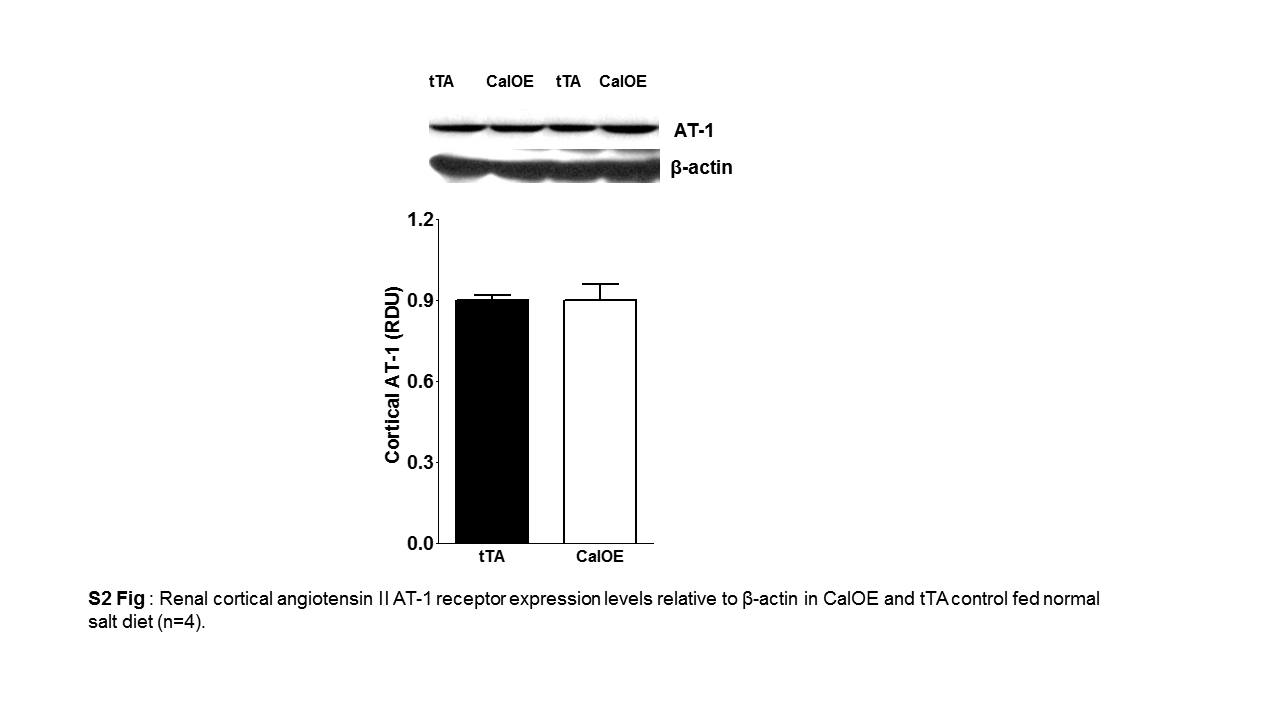

Supplement: S2 Fig — (TIF) [file pone.0211903.s003.TIF]

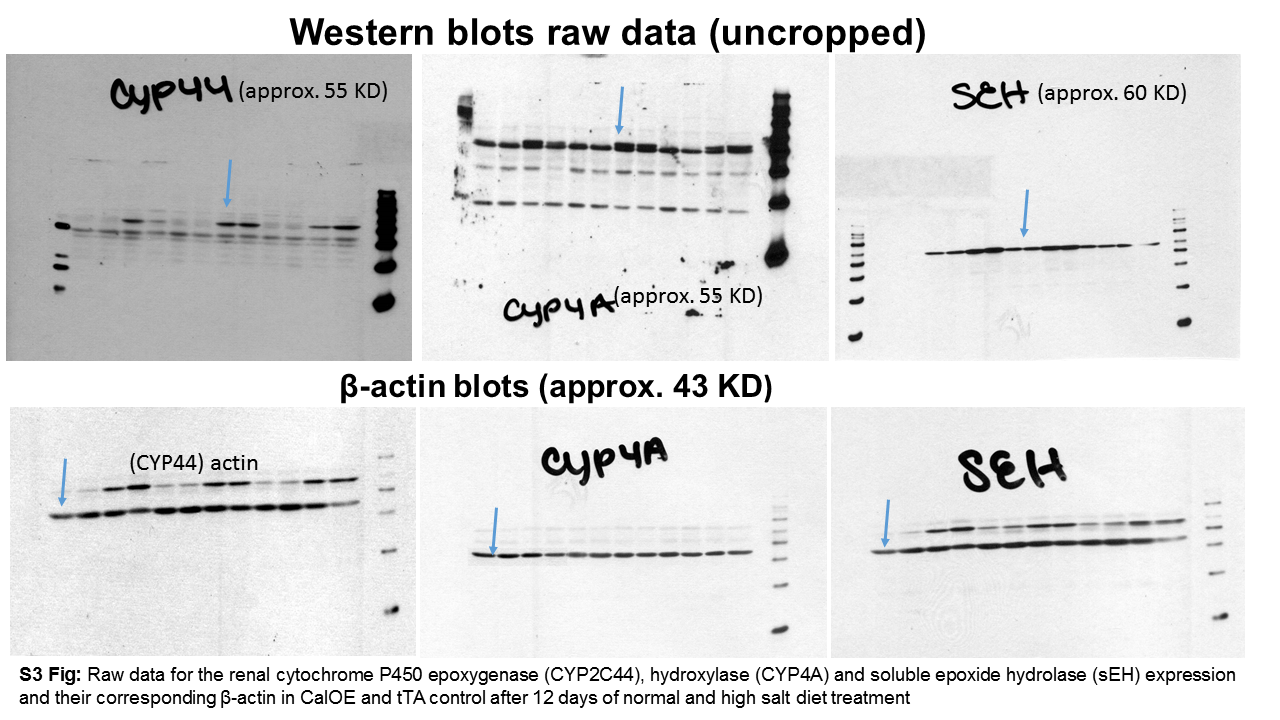

Supplement: S3 Fig — (TIF) [file pone.0211903.s004.TIF]
